# Staging recurrent ovarian cancer with ^18^FDG PET/CT

**DOI:** 10.3892/ol.2012.1075

**Published:** 2012-12-13

**Authors:** SANJA DRAGOSAVAC, SOPHIE DERCHAIN, NELSON M.G. CASERTA, GUSTAVO DE SOUZA

**Affiliations:** 1 DIMEN Medicina Nuclear and PET/CT Campinas, State University of Campinas-Unicamp, Campinas, São Paulo, Brazil;; 2 Departments of Obstetrics and Gynecology, State University of Campinas-Unicamp, Campinas, São Paulo, Brazil; 3 Radiology, Faculty of Medical Sciences, State University of Campinas-Unicamp, Campinas, São Paulo, Brazil

**Keywords:** ^18^FDG, PET scan, ovarian neoplasms

## Abstract

The aim of the present study was to evaluate the use of 2-deoxy-2-(^18^F)-fluoro-D-glucose (^18^FDG) positron emission tomography (PET)/computed tomography (CT) in patients with suspected ovarian cancer recurrence and describe the distribution of metastasis. A total of 45 female patients who underwent PET/CT scan due to raised CA-125 levels, clinical suspicion of ovarian cancer recurrence or alterations detected on ultrasound (US), CT or magnetic resonance imaging (MRI) were included in this retrospective study. PET/CT results were compared with histological findings (n=15) or clinical, laboratory and repeated imaging techniques during subsequent follow-up for at least six months (n=30). CA-125 was elevated in 34 patients, 14 patients had clinical symptoms of disease and 23 presented with alterations on US, CT and MRI. A total of 42 patients were confirmed to have ovarian cancer recurrence, all with abnormal findings on PET/CT. Three patients remained free of disease during clinical follow-up, all with normal PET/CT findings. There were 11 patients with raised CA-125 levels and normal conventional imaging, all with positive PET/CT. Among the 11 patients with normal CA-125 levels, eight presented with positive PET/CT scan. Lymph nodes were the most frequent site of relapse of disease, followed by peritoneal implants. Distant sites of metastasis included the liver, spleen, pleura, lung and bone. PET/CT detected unsuspected lesions in 20/45 patients (44.4%). ^18^FDG PET/CT was a useful tool for evaluating the extent of ovarian cancer recurrence. In the current series, lymph nodes were the most frequent site of relapse of disease, with supradiaphragmatic lymph node metastasis in a large number of cases.

## Introduction

Ovarian cancer accounts for 3% of all cases of cancer in females; however, it has the highest mortality of all gynecological cancers (1). Most patients have advanced stage disease at presentation due to a paucity and incidious onset of symptoms. Initial treatment consists of cytoreductive surgery and adjuvant platinum-based cytotoxic chemotherapy (1,2). Despite high response after initial treatment, 20–30% of patients with early-stage disease (stage IA–IIA) and up to 75% of patients with advanced disease (IIB–IV) present with recurrence within two years (2,3).

Imaging examinations, including ultrasound (US), computed tomography (CT) and magnetic resonance imaging (MRI), should be performed during patient follow-up when there is clinical suspicion of ovarian cancer recurrence or CA-125 elevation. A systematic review and meta-analysis by Gu *et al*(4) evaluated CA-125 levels, positron emission tomography (PET) alone, PET/CT, CT and MRI in diagnosing recurrent ovarian carcinoma. CA-125 levels had the highest pooled specificity (93%), while PET/CT had the highest pooled sensitivity (91%). CT (sensitivity, 79%; specificity, 84%) and MRI (sensitivity, 75%; specificity, 78%) had a similar diagnostic performance.

While the level of CA-125 has been shown to be a sensitive marker for tumor recurrence and levels may rise 3 to 6 months before there is clinically apparent disease, it does not provide information concerning the size and distribution of the lesions (3–5). Levels may also increase in a number of benign conditions, not being a specific marker for ovarian cancer, and a number of patients with relapse of disease present with normal CA-125 levels (4,5). CT has low sensitivity for detecting disease recurrence, probably due to its inability to detect small peritoneal implants and normal-sized lymph node metastases (4,6). Pelvic MRI is useful for the evaluation of local recurrence of disease, however, it has low specificity due to post-surgical anatomical alterations (4,7).

2-deoxy-2-(^18^F)-fluoro-D-glucose (^18^FDG) PET/CT may play an important role in ovarian cancer recurrence, as the metabolic tracer is able to increase lesion detection, the fusion of metabolic and anatomical imaging aids the determination of the exact location of disease and it is capable of surveying the whole body. Several studies have examined the performance of PET/CT scanning in patients with recurrent ovarian cancer (4,8–14).

An Australian prospective, multi-center cohort study of 90 females (14) assessed the impact of ^18^FDG PET/CT in the management of patients with suspected recurrent ovarian cancer and evaluated information provided by ^18^FDG PET/CT in this clinical context. PET/CT detected 168 additional and unsuspected sites of disease in 61 patients (67.8%). The management was changed in 53 patients (58.9%) based on PET/CT scan findings. PET/CT was superior to abdominal and pelvic CT in the detection of nodal, peritoneal and subcapsular liver disease and it also allowed the identification of patients whose disease was likely to progress within 12 months. The authors suggested that PET/CT should be the preferred imaging modality in patients with suspected ovarian carcinoma recurrence.

The aim of the present study was to evaluate the use of ^18^FDG PET/CT in patients with suspicion of ovarian cancer recurrence and describe the distribution of metastasis.

## Patients and methods

### Patients

A total of 45 female patients (age range, 39–84 years; mean age ± SD, 59.5±10.0) with suspicion of ovarian cancer recurrence were included in this retrospective study. The patients underwent a PET/CT scan at PET/CT Campinas, private clinic, Campinas, São Paulo, Brazil, between November 2006 and November 2010. Indications for PET/CT were clinical suspicion of relapse of disease, elevated CA-125 or abnormal or equivocal findings on abdominal/pelvic US, CT or MRI. All patients had undergone surgery and all but one had received adjuvant chemotherapy at the time of diagnosis. A total of 18 patients had already had relapse of disease during their previous follow-up and PET/CT was performed for the suspicion of new progression of disease. The study was approved by the ethics committee of the Medical Sciences Faculty, State University of Campinas, Unicamp, São Paulo, Brazil. Detailed patient and tumor characteristics are shown in Table I.

### ^18^FDG PET/CT imaging

All patients fasted for at least 6 h, maintaining their blood glucose levels <150 mg/dl, before the injection of ∼12 mCi (mean ± SD, 13.0±2.3 mCi) of ^18^FDG. The patients rested in a supine position for 40 to 60 min after the injection and were then positioned for PET/CT imaging. All PET/CT scans were performed on a combined 16-slice CT/BGO PET scanner (Discovery STE, GE Medical Systems Inc., Milwaukee, WI, USA). The patients received oral contrast (Urografina^®^ 291, Schering-Plough Corporation; Kenilworth, New Jersey, USA; 25 ml diluted in 1 liter of water), two glasses before the ^18^FDG injection and two glasses immediately before the imaging. A contrast-enhanced CT was acquired from the top of the head to mid-thigh, without any specific breath-holding instructions. Intravenous contrast (100 ml, Optiray, Mallinckrodt; St.Louis, MO, USA) was injected, unless the patient was allergic to iodine. The parameters of the CT scan were 140 kV, 150–250 mAs, slice thickness of 3.75 mm. The CT was followed by PET scanning, covering the same transverse field of view during normal breathing. The imaging was acquired with 6 to 8 bed positions on a 2D mode for 5 min per bed position (n=20). In August 2008, the protocol of the institution changed, therefore, the scans of 25 patients were acquired on a 3D mode for 3 min per bed position. PET images were reconstructed iteratively using the contrast-enhanced CT data for attenuation correction. Coregistered images were displayed on a workstation, using dedicated software which allowed the viewing of PET, CT and fusion images on transaxial, sagittal and coronal displays.

### ^18^FDG PET/CT analysis

All ^18^FDG PET/CT scans were interpreted by an experienced radiologist in conjunction with an experienced nuclear medicine physician, who were both aware of the suspicion of ovarian carcinoma recurrence and the laboratory and imaging findings of the patients. The ^18^FDG PET portion and the CT portion of PET/CT were jointly interpreted using a dedicated image fusion workstation. All areas of increased ^18^FDG uptake that corresponded to a CT abnormality were interpreted as positive for recurrent disease. Semi-quantitative analysis was also performed to derive a standardized uptake value (SUV). All PET/CT reports and images were reviewed by an experienced nuclear physician for consistency of the data.

The results of ^18^FDG PET/CT were correlated with patient follow-up information for at least 6 months after the examination (mean ± SD, 21.0±12.0). The diagnosis of recurrence was confirmed with surgery (n=15) or clinically (n=30), by persistent elevation of CA-125 levels with abnormal findings on further imaging and treatment response following chemotherapy.

### Statistical analysis

For the comparison of the SUVs from different tumor types the ANOVA test was used, and P<0.05 was considered to indicate a statistically significant difference.

## Results

A total of 42 patients were diagnosed with recurrence of ovarian cancer after surgery or during clinical follow-up. Three patients remained free of disease during clinical follow-up. CA-125 levels were raised in a total of 34 patients, 14 patients had clinical suspicion of recurrence and 23 presented with alterations on US, CT or MRI. There were 11 patients with raised CA-125 levels and normal imaging examinations. The characteristics of the patients according to the PET/CT findings are shown in Table II.

^18^FDG PET/CT scan was positive in all 42 patients who were confirmed to have recurrence of disease. ^18^FDG PET/CT scan was negative in 3 patients, all free from disease during follow-up, with normal CA-125 levels and no evidence of disease on imaging examinations. One of the patients without ovarian cancer recurrence presented with focal abnormal uptake in the right thyroid lobe (SUV, 17.0) and a new primary tumor was diagnosed following surgery.

**Figure 1.**
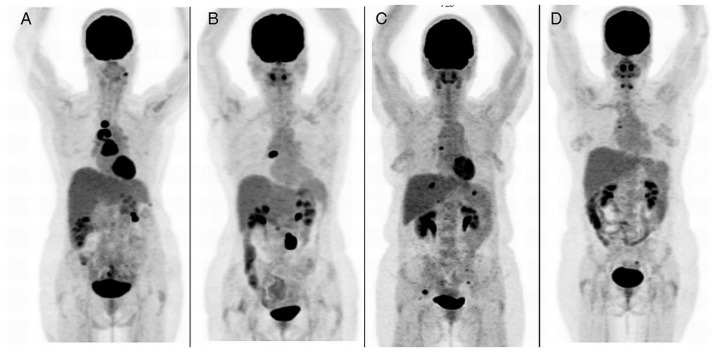
Patients with CA-125 levels within normal range with positive PET/CT scan showing supra-diaphragmatic lymph node and pelvic/abdominal metastases. CA-125 levels were (A) 13.8 U/ml; (B) 17.4 U/ml; (C) 20.8 U/ml; (D) 32.3 U/ml. PET, positron emission tomography; CT, computed tomography.

There were 11 patients with elevated CA-125 levels and normal conventional imaging, all with positive PET/CT findings. However, of the 11 patients with normal CA-125 levels, eight presented with a positive PET/CT scan (Fig. 1). Four of those patients had localized pelvic or abdominal disease (two of which were amenable to surgical resection) and 4 had supra-diaphragmatic lymph node metastasis. Patients with elevated CA-125 levels tended to have a more disseminated disease.

Overall, lymph nodes were the most frequent site of relapse of disease (Table III), being localized to the pelvic/abdominal region in 30 patients (66.7%) and the thoracic region in 16 (35.6%). Six patients had internal mammary metastasis, with surgical resection in two patients. Peritoneal implants were found in 27 patients (60%). Distant sites of metastasis included the liver (n=6), spleen (n=2), pleura (n=2), lung (n=2) and bone (n=2). However, with regard to the subgroup of 11 patients with elevated CA-125 levels and normal conventional imaging, peritoneal implants were the most frequent site of relapse of disease (n=9), followed by lymph nodes, most being in the pelvic/abdominal region (n=7).

**Figure 2.**
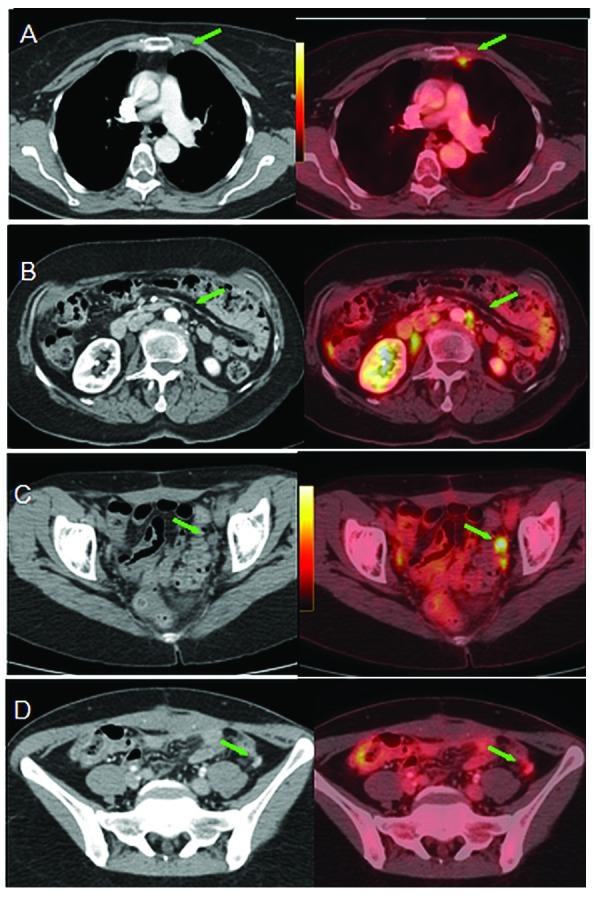
Unsuspected lesions detected with ^18^FDG PET/CT. The left column shows CT and the right column shows ^18^FDG PET/CT images. (A) Small hypermetabolic left internal mammary lymph node measuring 0.9 cm, with SUV=3.6 (green arrows). (B) Normal sized hypermetabolic abdominal paraaortic lymph node, <1.0 cm (green arrows). (C) Left iliac hypermetabolic lymph nodes (green arrows). (D) Small hypermetabolic implant in the left pelvic region, measuring 1.1 x 0.5 cm (SUV=1,5) (green arrows).

PET/CT found unsuspected lesions in 20 out of 45 patients (44.4%), most being supra-diaphragmatic lymph node metastases or normal sized abdominal lymph nodes with abnormal ^18^FDG uptake (Fig. 2). There was no statistical difference when comparing the SUVs of lesions from different tumor types (P=0.6683; Table IV).

A total of 12 patients (26%) died during follow-up (mean ± SD, 15.3±7.9 months after examination; range, 6–35 months). Five of these patients had disseminated abdominal and supra-diaphragmatic disease, while 7 had disease limited to the pelvic/abdominal region.

## Discussion

PET/CT correctly diagnosed patients with suspected ovarian cancer recurrence. All patients with elevated CA-125 levels and normal conventional imaging had positive PET/CT scan. However, most patients with normal CA-125 levels in this series presented with positive PET/CT scan. Lymph nodes were the most frequent site of relapse of disease, most being in the pelvic/abdominal region and others in the thoracic region. Peritoneal implants were found in more than half of patients. Distant sites of metastasis included the liver, spleen, pleura, lung and bone. PET/CT detected unsuspected lesions in almost half of the patients, most being supra-diaphragmatic lymph node metastasis.

Our population had a high pre-test probability of disseminated disease, given that 18/45 patients (40%) had previously had relapse of ovarian cancer and the referral to PET/CT was to evaluate the progression of the disease and to restage. The advantage of PET/CT in this clinical setting was ability to evaluate the whole body that may aid the correct selection of patients who are amenable to surgical resection.

Most of our findings are in accordance with those previously described in the literature, with the exception of the high prevalence of supra-diaphragmatic lymph node metastases (7–14). Iagaru *et al*(9) retrospectively evaluated 43 patients with ovarian carcinoma and described the distribution of extra-pelvic metastases in 19 patients, 5 of which (11.6%) had supra-diaphragmatic lymph node involvement. A prospective, multi-center Australian study (14) showed that 14 out of 90 patients (15%) presented with disease above the diaphragm.

In the Australian study (14), PET/CT findings changed the management plan with medium to high impact in 58% of patients, due to its ability to detect unsuspected lesions and the advantage of the whole body evaluation.

The change in management based on PET/CT was also previously described by Simcock *et al*(8). The authors prospectively evaluated 56 females with ovarian cancer who underwent PET/CT scan for suspicion of recurrence or surveillance with no evidence of diseacse. PET/CT altered the apparent disease distribution in 40 scans (61%), with a high impact on management plan in 32 patients (57%).

Numerous clinicians routinely measure the level of CA-125 since it is often the first evidence of ovarian cancer recurrence and may rise 3 to 6 months before clinical evidence of disease (3–5). However, without localized disease, there is no rationale to initiate treatment based on a laboratory test alone and this can cause considerable patient anxiety. Therefore, it is important to have an accurate method that is able to both diagnose and localize the recurrence of disease and aid the selection of patients amenable to surgical resection.

Recent meta-analyses evaluated CT, MRI, PET and PET/CT for the detection of metastatic lymph nodes in patients with ovarian cancer (15). PET and PET/CT were a more accurate modality for lymph node metastasis detection, with a global pooled sensitivity of 73.2% and a specificity of 96.7%. However, the greater specificity of PET or PET/CT compared with those of CT or MRI was statistically insignificant. CT and MRI showed similar diagnostic performance, with pooled sensitivity of 42.6 and 54.7% and pooled specificity of 95.0 and 88.3%, respectively.

PET/contrast-enhanced CT in the same study (15) showed a sensitivity of 84.4% and specificity of 97.4%, which was better than non-contrast-enhanced PET/CT. Certain authors believe that the CT portion of the study should be performed without contrast (8). Those who defend the use of contrast usually perform a non-contrast-enhanced CT before the diagnostic contrast-enhanced CT, for the purposes of attenuation correction of the PET images. Yau *et al*(16) showed that application of intravenous contrast does not interfere with the diagnostic value of PET/CT when contrast-enhanced CT is used for attenuation correction purposes.

The PET/CT evaluation of pelvic and abdominal regions may be challenging due to urinary excretion and bladder concentration of ^18^FDG. Contrast material may aid the distinguishing of vessels and urethers from small nodal disease, which can result in better sensitivity of the PET/CT scan. This may be of particular importance in patients with ovarian cancer, since most metastases involve the pelvic and abdominal lymph nodes or implants. Certain authors (17) suggest the use of diuretics and dual time imaging as another way of improving the sensitivity of the examination for detection of pelvic lesions.

A limitation of our study is that there was no pathological confirmation of all the sites of abnormal ^18^FDG uptake. However, the confirmation of all the sites would not have been ethical solely for the purpose of validation of PET/CT findings. Accurate surgical assessment of pelvic and retroperitoneal lymph nodes is difficult, and surgery appears to be an unreliable gold standard, with disease recurrence in a third of females with negative surgical findings (18). We agree with Simcock *et al*(8) and believe that the course of disease and clinical outcomes may more accurately validate PET/CT data.

Another limitation was that we did not have data concerning the treatment plans prior to the PET/CT, therefore, it was not possible to evaluate the change in management in our study. However, PET/CT revealed unsuspected lesions in 44.4% of our patients, which is in accordance with previously published data.

There is no evidence that PET/CT improves the overall survival of patients diagnosed with ovarian cancer recurrence. However, the whole body examination shows the extent of the disease. This may aid the correct restaging of patients considered for further treatment.

In conclusion, ^18^FDG PET/CT was an accurate and useful tool for diagnosing ovarian cancer recurrence. The advantage of a whole body scan and metabolic imaging is that it may aid the detection of additional sites of disease. Supra-diaphragmatic disease in this series of patients with suspicion of ovarian cancer recurrence was more frequent than previously described.

## Figures and Tables

**Table I.** Patient and tumor characteristics (n=45).

Characteristic	Value
Age (years)	
Mean ± SD	59.5±10.0
Range	39–84
Histological type (n)	
Serous adenocarcinoma	29
Endometrioid adenocarcinoma	9
Mixed type adenocarcinoma	1
Clear cell adenocarcinoma	1
Adenocarcinoma (NOS)	4
Sertoli cell tumor	1
FIGO stage at diagnosis (n)	
I	2
II	3
III	34
IV	6

NOS, not otherwise specified; FIGO, International Federation of Gynecology and Obstetrics.

**Table II.** Characteristics of patients according to PET/CT findings.

	PET/CT	
Characteristic	Positive	Negative
Recurrent disease		
Yes	42	-
No	-	3
CA-125 (U/ml)		
>35	34	-
≤35	8	3
Clinical symptoms		
Positive	14	-
Negative	18	1
No data	10	2
US		
Positive	4	-
Negative	6	1
No data	32	2
MRI		
Positive	4	-
Negative	5	-
No data	33	3
CT		
Positive	13	2
Negative	10	1
No data	19	-
Surgery after PET/CT		
Positive	15	-
Negative	-	-
Not performed	27	3

PET, positron emission tomography; CT, computed tomography; US, ultrasound; MRI, magnetic resonance imaging.

**Table III.** Distribution of metastases found on PET/CT.

		SUV (mean ± SD)	
Localization	Number of patients	Normal CA-125 (n=8)	Elevated CA-125 (n=34)
Pelvic and abdominal lymph nodes	30	6.1±4.0	6.9±4.6
Thoracic lymph nodes	16	11.3±4.3	4.8±3.0
Peritoneal implants	27	7.5±3.1	6.8±3.8
Liver	5	-	7.6±4.0
Spleen	2	-	9.0±5.3
Pleura	2	-	4.4±4.9
Lung	2	-	1.7±0.1
Bone	2	-	2.8±0.8
Thoracic wall implants	1	5.7±0.0	-

SUV, standardized uptake value; PET, positron emission tomography; CT, computed tomography.

**Table IV.** SUV of the lesions among different cancer types (P=0.6683).

Histological type of tumor	Number of lesions	SUV (mean ± SD)
Serous adenocarcinoma	133	6.8±4.3
Endometrioid adenocarcinoma	29	6.1±3.7
Mixed type adenocarcinoma	3	3.9±0.2
Clear cell adenocarcinoma	3	4.5±1.7
Adenocarcinoma (NOS)	15	5.2±2.9
Sertoli cell tumor	3	7.5±4.0

SUV, standardized uptake value; NOS, not otherwise specified.
